# Cytotoxicity, fluorescence tagging and gene-expression study of CuInS/ZnS QDS - meso (hydroxyphenyl) porphyrin conjugate against human monocytic leukemia cells

**DOI:** 10.1038/s41598-020-61881-8

**Published:** 2020-03-18

**Authors:** Ncediwe Tsolekile, Sara Nahle, Nkosingiphile Zikalala, Sundararajan Parani, El Hadji Mamour Sakho, Olivier Joubert, Mangaka C. Matoetoe, Sandile P. Songca, Oluwatobi S. Oluwafemi

**Affiliations:** 1 Department of Chemical Sciences, University of Johannesburg, P. O. Box 17011, Doornfontein, 2028 Johannesburg, South Africa; 2 Centre for Nanomaterials Science Research, University of Johannesburg, Johannesburg, South Africa; 3 Department of Chemistry, Cape Peninsula University of Technology, P.O. Box 652, Cape Town, 2000 South Africa; 4 Université De Lorraine, Faculté de Pharmacie, F-54001 Nancy Cedex, France; 5 Department of Chemistry, University of KwaZulu-Natal, Private Bag X 54001, Durban, 4000 South Africa

**Keywords:** Biotechnology, Nanobiotechnology

## Abstract

The toxicity of heavy metals present in binary semiconductor nanoparticles also known as quantum dots (QDs) has hindered their wide applications hence the advent of non-toxic ternary quantum dots. These new group of quantum dots have been shown to possess some therapeutic action against cancer cell lines but not significant enough to be referred to as an ideal therapeutic agent. In this report, we address this problem by conjugating red emitting CuInS/ZnS QDs to a 5,10,15,20-tetrakis(3-hydroxyphenyl)porphyrin -photosensitizer for improved bioactivities. The glutathione capped CuInS/ZnS QDs were synthesized in an aqueous medium using a kitchen pressure cooker at different Cu: In ratios (1:4 and 1:8) and at varied temperatures (95 °C, 190 °C and 235 °C). Optical properties show that the as-synthesized CuInS/ZnS QDs become red-shifted compared to the core (CuInS) after passivation with emission in the red region while the cytotoxicity study revealed excellent cell viability against normal kidney fibroblasts (BHK21). The highly fluorescent, water-soluble QDs were conjugated to 5,10,15,20-tetrakis(3-hydroxyphenyl)porphyrin (mTHPP) via esterification reactions at room temperature. The resultant water-soluble conjugate was then used for the cytotoxicity, fluorescent imaging and gene expression study against human monocytic leukemia cells (THP-1). Our result showed that the conjugate possessed high cytotoxicity against THP-1 cells with enhanced localized cell uptake compared to the bare QDs. In addition, the gene expression study revealed that the conjugate induced inflammation compared to the QDs as NFKB gene was over-expressed upon cell inflammation while the singlet oxygen (^1^O_2_) study showed the conjugate possessed large amount of ^1^O_2_, three times than the bare porphyrin. Thus, the as-synthesized conjugate looks promising as a therapeutic agent for cancer therapy.

## Introduction

The use of nanomaterials for biological application has in the past decade transpired as a promising avenue to explore for biological application due to their size, shape, specific surface area, aspect ratio and surface chemistry. Of the reported nanomaterial, semiconductor nanomaterials also known as quantum dots (QDs) still remain the most promising due to their excellent optoelectronic properties and spectral tunability which allows for their use in the ultra-violet and near infra-red region^1,2^. These inherent properties have made QDs more appealing compared to organic fluorophores and have led to their use in a variety of bio- applications such as bio-imaging, diagnostics, drug delivery, *in-vitro* single molecule sensing and *in-vivo* cell tracking^3–5^. Most of the reported studies on QDs have focused on binary semiconductor nanocrystals with many advances being reported with regards to their synthesis and application^6,7^. Despite the vast amount of work reported on the application of binary QDs by various groups^8,9^, the clinical use of the binary QDs has been limited by the presence of toxic heavy metals (Cd, Pb, Hg) in their composition.

This has prompted the investigation of less-toxic alternative materials composed of group I (Cu, Ag), group III (In, Ga, Al) and group VI (S, Se, Te) elements called “ternary” quantum dots. Amid these QDs, much attention has been given to CuInS based QDs^10^. Interest in ternary QDs lies in their reduced toxicity; size- and composition – tunable photoluminescence properties^11–13^, high Stokes shift and long fluorescence lifetime all which has legitimated their use in biological applications such as biomedical imaging and drug delivery^14,15^. The conjugation of nanomaterials such as quantum dots (QDs) to porphyrins has been reported as an improvement on the biological application of porphyrins in treatment modalities such as photodynamic therapy (PDT) where porphyrins have been most applicable. Cosme *et al*.^16^ recently reported on the use of carbon dot-protoporphyrin IX conjugates for improved drug delivery and bio-imaging. Yang *et al*.^17^ reported on the targeting ability and subcellular localization of folate-conjugated platinum porphyrin complex (Por 4). The group reported that, modification of the carboxyl group with a porphyrin compound, did not decrease the binding affinity of folic acid to folate receptors (FR) positive cancer cells. They obtained 2 2% and 75% 20 Μm cell viability for HeLa and A549 cells, respectively at 20 μM. Recently our group has also reported reported on the conjugation of meso-tetra-(4-sulfonatophenyl) porphyrin (TPPS_4_) – CuInS/ZnS QDs conjugate as an improved photosensitizer with enhanced singlet oxygen generation of the porphyrin from 0.19 for TPPS_4_ alone to 0.69 upon conjugation^18^. Noteworthy is the verity that most cytotoxicity reports on CuInS based QDs focus their studies on cancer cell lines, neglecting the significance of reporting the selectivity/therapeutic index of the material with regards to healthy and tumour/cancer cells. Additionally, the reported cytotoxicity values are not adequate enough for the CuInS/ZnS QDs to be referred to as an ideal therapeutic agent. Moreover, synthetic disadvantage to the ternary QDs is the blue-shifted emission commonly reported during the growth of ZnS over CuInS based QDs^19,20^. This has affected their fluorescence properties and to date, the exact mechanism to explain the blue-shift is still under review with many researchers attributing it to the cation exchange of Cu^+^ and In^3+^ with Zn^2+^ or the inter-diffusion of Zn atoms into the core during surface passivation^1^. In this paper, we report on large-scale synthesis of glutathione-capped-CuInS/ZnS QDs. Temporal evolution of the optical properties of the core and core-shell QDs was investigated by varying the Cu: In ratio (1: 4 and 1: 8) and the reaction temperature (95 °C, 190 °C and 235 °C). The optimized condition for the synthesis was at ratio 1:4 and 95 °C with bathochromic shift emission from 525 nm for the core to 607 nm for the core/shell. To the best of our knowledge, this red shifting after the shell formation has never been reported for aqueous synthesized ternary CuInS based QDs. Cell viability studies of the CuInS/ZnS QDs on normal kidney fibroblast cell line (BHK21) showed the material possess excellent viability towards healthy cells. This prompted the investigation of cytotoxicity of the material against a human monocytic leukaemia cell model (THP-1) whereby dose dependent cytotoxity was obtained for the QDs. To improve on the therapeutic abilities of the as-synthesized GSH capped CuInS/ZnS QDs, we conjugated the QDs to 5,10,15,20-tetrakis(3-hydroxyphenyl)porphyrin (mTHPP). The conjugate was obtained via esterification reaction to produce QD-porphyrin conjugate with good water solubility and optical properties. In this report, the as-synthesized conjugate was tested against THP-1 via a series of *in vitro* studies which included cytotoxicity using WST-1 cell proliferation assay, bio-labelling and gene expression using reverse transcription (RT) and quantitative real-time RT-polymerase chain reaction (qRT-PCR). Based on the good results obtained from the *in vitro* study against the leukaemia cell line, the singlet oxygen generation (SOQY) ability of the conjugate was also evaluated.

## Results and Discussion

In this study, CuInS and CuInS/ZnS QDs were synthesized in large scale using a pressure cooker. The core material was synthesized by heating CuInS precursors in the presence of glutathione (GSH) and sodium citrate as dual stabilizers to counter the different reactivity’s of the cation precursors based on the Pearson’s hard/soft acid/ base principle^21^. This was then followed by the shell growth. Glutathione served as a stabilizing and reducing agent for Cu^2+^ ions and to enhance the bio-compatibility of the QDs. The precursor molar ratios and the effect of temperature on the optical properties of the as-synthesized ternary QDs were studied and their optical properties were evaluated using photoluminescence (PL) and ultraviolet –visible (UV-VIS) spectrophotometer.

### Effect of the Cu: In ratio on the optical properties of CuInS and CuInS/ZnS QDs

**Figure 1:**
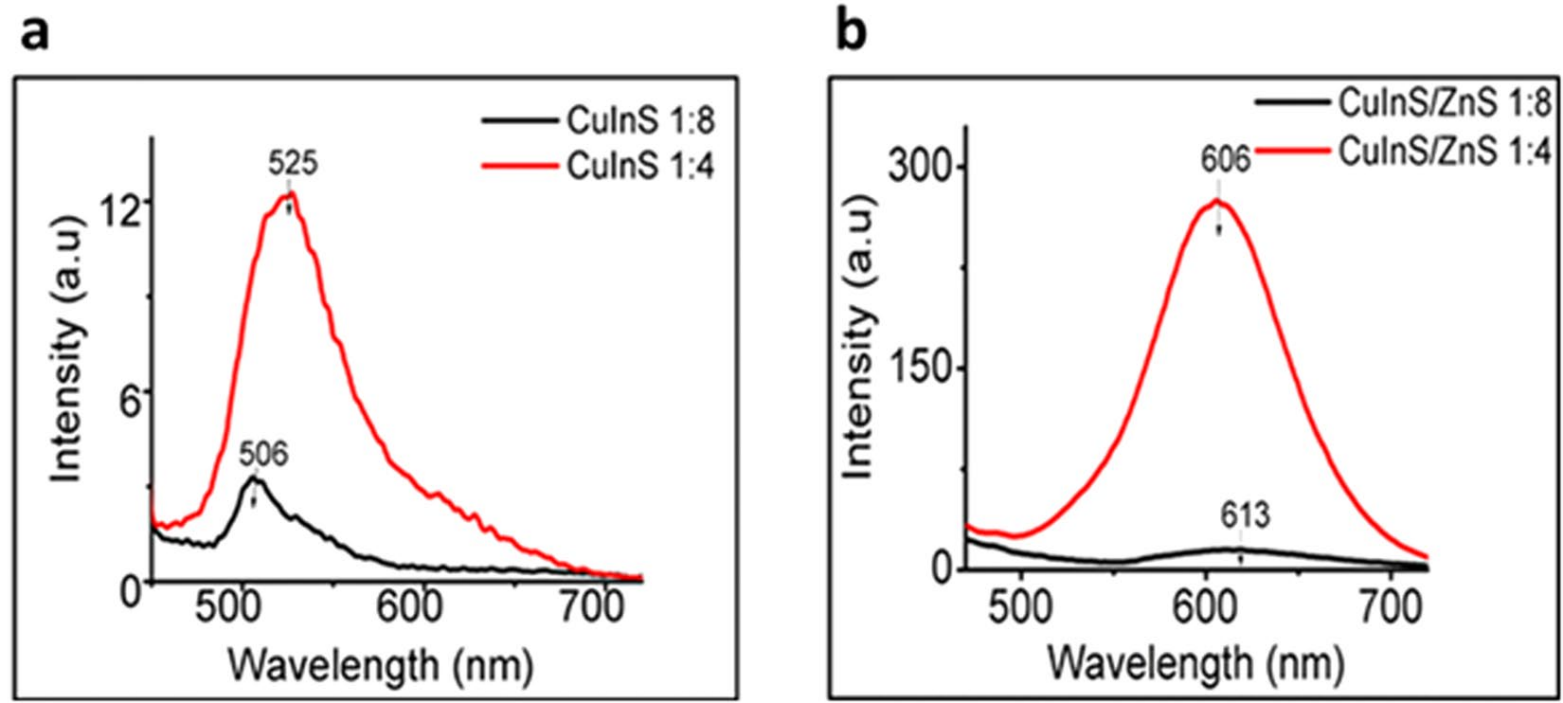
PL emission spectra of (**a**) CuInS QDs and (**b**) CuInS/ZnS QDs synthesized at Cu: In ratio of 1:4 and 1:8 at 95 °C.

Figure 1a,b shows the PL spectra at two different feeding Cu: In ratio’s (1:4 and 1:8) synthesized at 95 °C. The PL emission of the CuInS QDs was significantly modified by the Cu: In ratio. Increasing the Cu: In ratio from 1:4 to 1:8, resulted in reduced intensity and significant blue-shifting of the PL peak from 525 nm to 506 nm (Fig. 1a). This indicates that growth at lower Cu concentration was characterized with the generation of intrinsic defects such as Cu interstitials (Cu_i_), Cu-to-In antisites (Cu_In_), and In vacancies (V_In_) which reduced the emission intensity. Similar blue-shifted emissions are always reported at lower Cu ratio^22,23^. To address the surface defects and improve the luminescent intensity, ZnS shell passivation was applied, which resulted in red-shifted narrow emissions at both Cu: In ratio’s (Fig. 1b) with improved luminescent intensity. The red-shifted emission after shell formation is attributed to the dominance of quantum confinement effect arising from particle size variation over cation exchange process- most common mechanism for the blue –shifted emission after the shell growth. The Zn: Cu + In ratio during shell passivation was kept constant at 2.5: 1, which proved to be adequate and effective in addressing the surface defects, and subsequently passivate the core effectively thus, preventing slight etching of the CuInS core size during the shell growth.

### Temperature dependent optical properties of CuInS and CuInS/ZnS QDs

**Figure 2:**
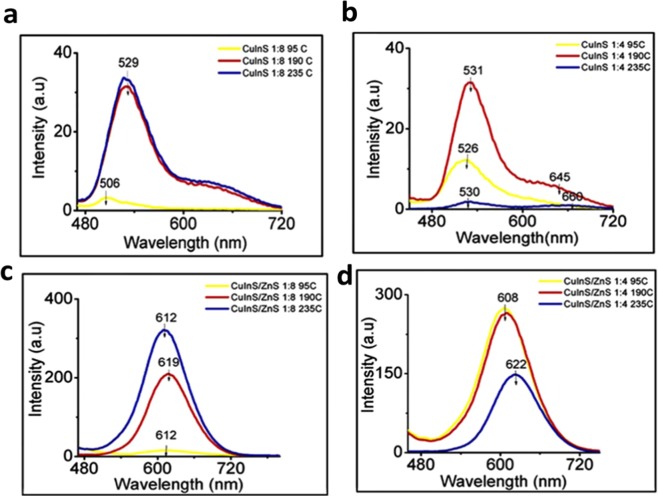
PL emission spectra of (**a**) CuInS QDs at Cu: In (1:8), (**b**) CuInS QDs at Cu: In (1:4), (**c**) CuInS/ZnS QDs at Cu: In (1:8) and (**d**) CuInS/ZnS QDs at Cu: In (1:4) synthesized at different temperature of 95 °C, 190 °C and 235 °C.

The crystal structure, quality and optical properties of CuInS QDs can be modified by varying reaction temperature due to the large degree of freedom in the composition and lattice structure of CuInS QDs^24^. For large scale synthesis, low temperatures are ideal with direct aqueous synthesis usually performed at 90–95 °C temperature range^17,24,25^. In this study, CuInS core and CuInS/ZnS core/shell were synthesized at 95 °C, 190 °C and 235 °C. Supplementary Fig. S1 shows the UV absorption of CuInS QDs core and CuInS/ZnS QDs core/shell synthesized at 1:4 at different synthetic temperatures. From Fig. 2a,b, multi-peak PL emissions with peak intensities dependent on the Cu: In ratio and temperature are observed. Consequently, peak 1 refers to the peak of highest intensity (left-side peak) and the other peak towards longer wavelengths is referred to as peak 2. The two peaks are attributed to two different trap states surface and intrinsic trap states characteristic of ternary QDs^26,27^. Due to the small size of the CuInS QDs, few defects or impurity phases could introduce a great difference in the optical properties of the QDs. This is apparent in the increased intensity of peak 2 and the shift in the position of both peaks with changes in temperature. Peak 2 becomes more defined at high temperatures, particularly at 1:8, Cu:In ratio (Fig. 2a), where an increase in temperature resulted in an increase in intensity contrary to 1:4 (Fig. 2b where the PL intensity increased initially and declined afterwards. The reduction in PL intensity is attributed to the principle that at low temperatures, carriers can thermally escape to surface defect states, which are non-radiative recombination centres, resulting in the reduction of PL intensity^28^. Upon ZnS shell passivation, a similar trend in PL intensity enhancement is observed with increase in temperature at 1:8 (Fig. 2c). The PL peak positions of the CuInS/ZnS become red-shifted from the CuInS core peak 1 with improved intensity. Figure 2d shows that the ZnS passivation of the CuInS core at 1:4 also resulted in PL enhancement at reduced reaction temperatures with red shifted emissions attributed to proper passivation of the surface defects and the relaxation of quantum confinement conditions.

### Structural and elemental composition

**Figure 3:**
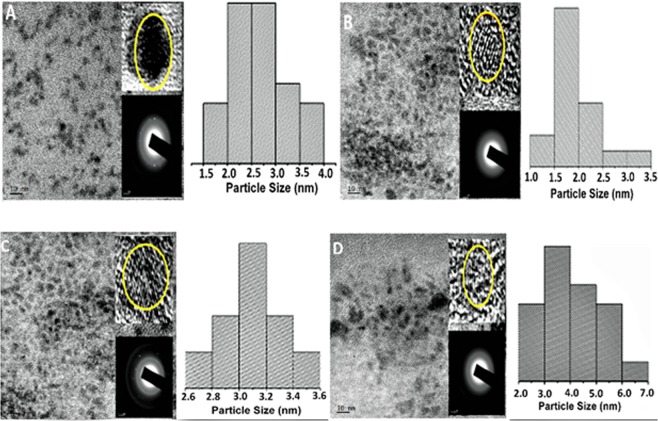
TEM images and size distribution curves of CuInS QDs core at (**a**) 1:8, (**b**) 1:4 and CuInS/ZnS QDs core/shell at (**c**) 1:4 and (**d**) 1:8 synthesized at 95 °C (insets: HRTEM and SAED patterns).

The morphology of the CuInS QDs and CuInS/ZnS QDs were studied using TEM and HRTEM. Figure 3a–d shows the TEM images of the as-synthesized material at the different Cu: In ratio at 95 °C. The micrographs show that the as-synthesized material are small, monodispersed and spherical in shape. The presence of the lattice fringes in the HRTEM and singular reciprocal point arranged in a ring in the selected area electron diffraction (SAED) pattern (Fig. 3a–d inset) further confirm the nano-crystalline nature of the as-synthesized materials. The average particle diameter, as determined from the TEM images and particle size distribution curve are 3.1 nm (1:8) and 2.1 nm (1:4) for the core CuInS QDs which increased to 3.2 nm (1:8) and 4.1 nm (1: 4) after the shell passivation.

**Figure 4:**
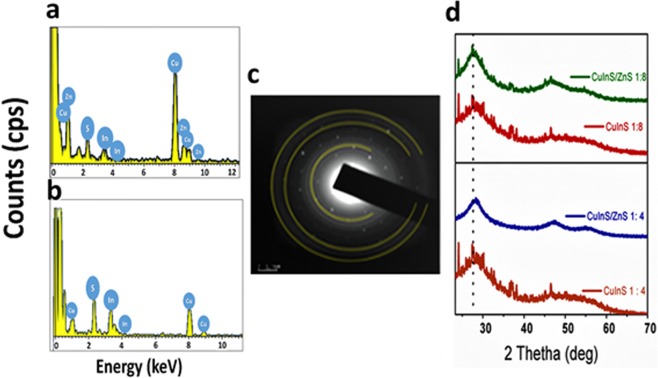
EDS spectra of (**a**) CuInS QDs (**b**) CuInS/ZnS QDs at ratio (1:4), (**c**) XRD diffraction pattern of CuInS/ZnS QDs synthesized at 95 °C and (**d**) SAED pattern of CuInS/ZnS QDs.

A representative EDS spectrum displaying the elemental composition of the CuInS core and CuInS/ZnS core/shell is shown Fig. 4a,b respectively. The EDS spectrum shows the presence of Cu, In and S only in the core CuInS QDs and an additional Zn peak in addition to the Cu, In and S in the core-shell. XRD measurements were performed using a Scintag XGEN-4000 ×-ray diffractometer with a CuKα (λ = 0.154 nm) radiation source. Figure 4c depicts the XRD patterns of CuInS core (1:4 and 1:8) and CuInS/ZnS core/shell (1:4 and 1:8) synthesized at 95 °C. The XRD patterns of the XRD CuInS core are typical of the standard CuInS (JCPDS 27–0159). After shell passivation, the peaks shifted to higher angle as a result of the ZnS passivation with diffraction peaks corresponding to CuInS and ZnS (JCPDS 05-0566) zinc blende crystal phases^29,30^. The broad nature of the diffraction pattern is attributed to the nano-crystalline nature of the material. This further supports the small size of the particles as observed in the TEM micrograph. The SAED pattern of the core-shell (Fig. 4d) gave three rings corresponding to the XRD diffraction patterns.

**Figure 5:**
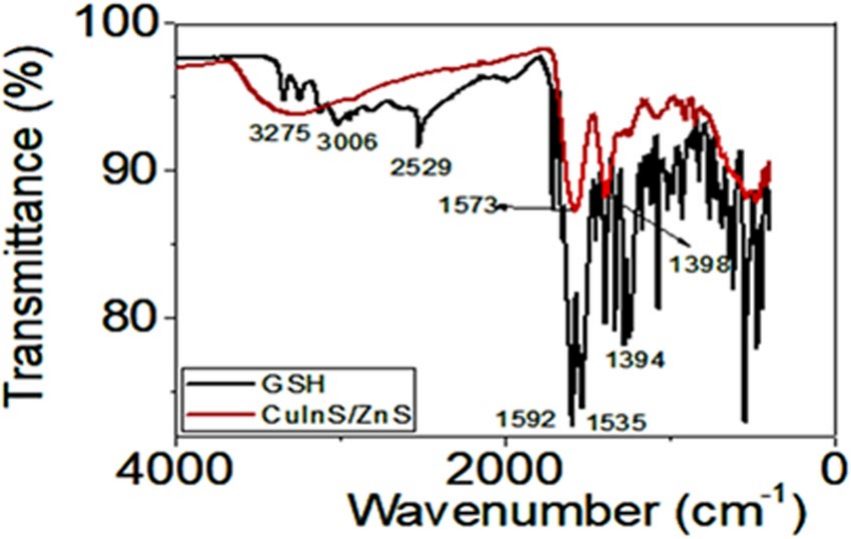
FTIR spectra of glutathione and glutathione capped-CuInS/ZnS QDs.

Figure 5 shows a representative FTIR spectra of the pure glutathione (GSH) and GSH-capped CuInS/ZnS QDs. The pure GSH shows peaks at 1394 cm^−1^, 3121 cm^−1^ and 3006 cm^−1^ assigned to the C–O stretching and N–H stretching vibration of the zwitterion–OOC–C–NH^3+^. The peaks at 1592 cm^−1^ and 1709 cm^−1^ are assigned to the N-H deformation and the C=O stretching band of the carboxylic group (ν C=O). The bands at 1535 cm^−1^ and 2529 cm^−1^ are assigned to -NHR and −SH, respectively. The spectrum for GSH capped CuInS/ZnS QDs, shows band at 3275 cm^−1^ assigned to the -O-H group. A shift to lower wavelength is observed for the stretching vibrations of C=O and C-NH bending vibrations to 1573 cm^−1^ and 1398 cm^−1^ respectively. The complete disappearance of the –SH stretching vibrational peak in the CuInS/ZnS suggest coordination of the -SH with metal atom and confirm the capping of CuInS/ZnS by GSH^31^.

### Characterization of 5,10,15,20-tetrakis(3-hydroxyphenyl)porphyrin (mTHPP) and CuInS/ZnS-mTHPP conjugates

**Figure 6:**
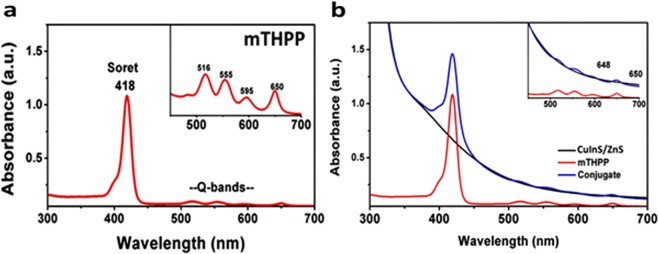
UV absorption of (**a**) mTHPP and (**b**) CuInS/ZnS QDs, mTHPP and CuInS-mTHPP conjugate.

5,10,15,20-tetrakis(3-hydroxyphenyl)porphyrin (mTHPP) was synthesized and conjugated to the red-shifted CuInS/ZnS QDs. The conjugate formation is attributed to the susceptibility of the carboxylic group of the GSH capped CuInS/ZnS to esterification reaction with the hydroxyl group of mTHPP porphyrin. Subsequently, strong magnetic stirring at room temperature proved sufficient to form a stable water soluble CuInS/ZnS – mTHPP conjugate via the conjugation of the -OH and COOH dangling groups on the porphyrin and QDs respectively. The as-synthesized mTHPP gave the characteristic Soret (418 nm) and Q-bands (516, 555, 595, 650 nm) (Fig. 6a). After the conjugation, the conjugate maintained the spectroscopic properties of both the CuInS/ZnS QDs and mTHPP as shown in Fig. 6b. The presence of all the Q-bands in the conjugate indicates that the mTHPP remained unmetallated upon conjugation.

**Figure 7:**
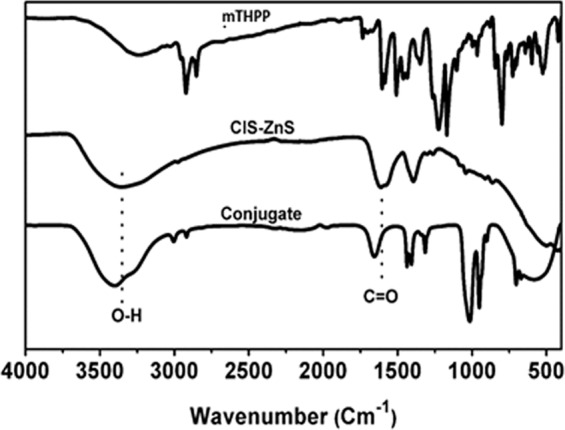
FTIR spectra of mTHPP–CuInS/ZnS QDs and the conjugate. Cell viability study of CuInS/ZnS QDs against BHK21 cancer cell line.

Excitation of the mTHPP and the conjugate at different wavelengths resulted in a hypochromic effect on the emission peaks of porphyrin (655 nm and 725 nm) as shown in Supplementary Fig. S2. The introduction of additional groups to change the charge redistribution, varying of electron donating or accepting strength of terminal groups and the elongation of the π-conjugation length amongst other factors have been reported to enhance two photon absorption behaviour of porphyrins^30^. In this study, the ability of the as-synthesized mTHPP and conjugate to emit at wavelengths (655 and 725 nm) lower than excitation wavelength (800 nm) suggests that the material has two photon absorption properties and thus suitable for biological applications such as PDT and photo-thermal therapy (PTT). The decrease in absorbance and slight blue-shift of the conjugate (Fig. 6b) relative to absorption of the bare porphyrin indicates the formation of the conjugate. This shows that the CuInS/ZnS core/shell has spectroscopic effect on the porphyrin suggesting a transfer in energy between the QDs and the mTHPP porphyrin. To further support the formation of conjugate via esterification, FTIR analysis was performed on the conjugate as shown in Fig. 7. From the FTIR, it can be seen that the mTHPP N-H pyrrole bands at 2854–2923 cm^−1^ are slightly red-shifted to 2920–3000 cm^−1^ in the conjugate. The disappearance of the 1509 cm^−1^ NH_2_ bending of the mTHPP, C=C and C=N vibrations at 1509–1605 cm^−1^ bands and the formation of the intense C=O band at 1015 cm^−1^ in the conjugate confirmed the formation of the conjugate.

### Biological application of CuInS/ZnS and CuInS/ZnS-mTHPP conjugate

**Figure 8:**
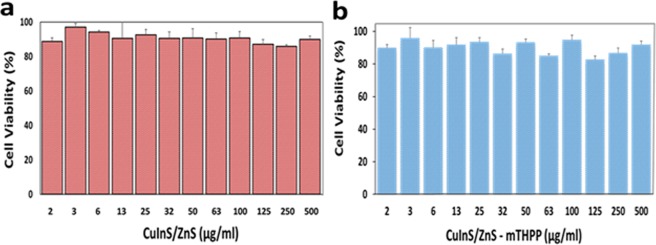
Cell viability study of (**a**) CuInS/ZnS QDs and (**b**) CuInS/ZnS-mTHPP conjugate QDs against BHK21 normal cell line. Cytotoxicity studies of (**a**) CuInS/ZnS QDs and (**b**) CuInS/ZnS – mTHPP conjugate against THP-1 cancer cell line.

Cell viability studies of CuInS/ZnS on normal fibroblast cancer cell line (BHK21) were determined using MTS assay. The BHK21 was exposed to varied concentrations of the QDs and conjugate (1.6 µg/ml–500 µg/ml) and the cell viability was measured across two plates in triplicates (n = 6). As shown in Fig. 8, the cell viability on the BHK21 was above 82%, even at high concentrations for both QDs and its conjugate. The remarkable cell viability of the as-synthesized material on normal cells shows that material is conducive for *in-vitro* and *in-vivo* biological application.

### Cytotoxicity study of CuInS/ZnS and CuInS/ZnS – mTHPP conjugate on leukaemia cancer cell line (THP-1)

**Figure 9:**
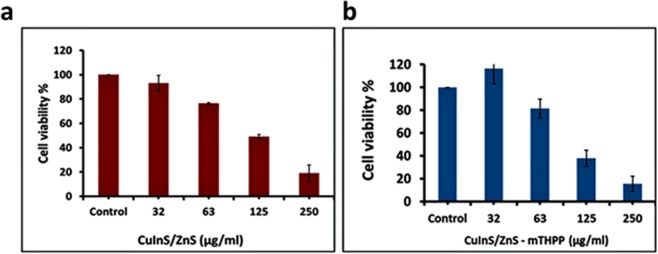
Cytotoxicity studies of (**a**) CuInS/ZnS QDs and (**b**) CuInS/ZnS – mTHPP conjugate against THP-1 cancer cell line. Confocal images of THP-1 cancer cell line under control (A–C), CuInS/ZnS QDs (D–F) and CuInS/ZnS-mTHPP conjugate (G–I) QDs for 12 h (scale = 20 µm).

The excellent cell viability results of the CuInS/ZnS QDs against normal cell prompted us to assess the selective/therapeutic index of the material by evaluating its cytotoxicity against leukaemia cancer cell line (THP-1). The THP-1 cell line was exposed to increasing concentrations of CuInS/ZnS QDs and a colorimetric assay, WST -1 was used to calculate the cell viability of the QDs after exposure. WST 1 was used due to its ability to produce water soluble cleavage products. It measures mitochondrial activity by giving different absorption spectra of formazan formed by reduction of tetrazolium by mitochondrial dehydrogenases. As seen from Fig. 9a, a dose dependent cytotoxicity was obtained, significant from 63 µg/ml for the CuInS/ZnS QDs core/shell (Cu: In (1:4) synthesized at 95 °C). This suggests that the CuInS/ZnS QDs is selective towards its effect on different types of cells (i.e. normal cells vs cancer cells) as it showed little nor no toxicity towards normal healthy cells but some toxicity towards the cancer cells thereby signifying a probable high selective index. To improve the therapeutic efficacy of the QDs, it was conjugated to mTHPP porphyrin and *in-vitro* cytotoxicity studies of the conjugate was performed on leukaemia THP-1 cancer cell line. As seen from Fig. 9b, the conjugate shows a significant cytotoxicity against the THP-1 at 125 µg/ml (reduction of <40%) compared to the quantum dots alone. The improved cytotoxicity of the conjugate is attributed to the combined effect of QDs and porphyrin resulting in improved therapeutic efficiency of the QDs. This substantiating the hypothesis that conjugating ternary QDs with porphyrins such as mTHPP could be the key to cancer treatment and thus establishing ternary CuInS/ZnS QDs – porphyrin conjugates as therapeutic agents.

### Confocal imaging

**Figure 10:**
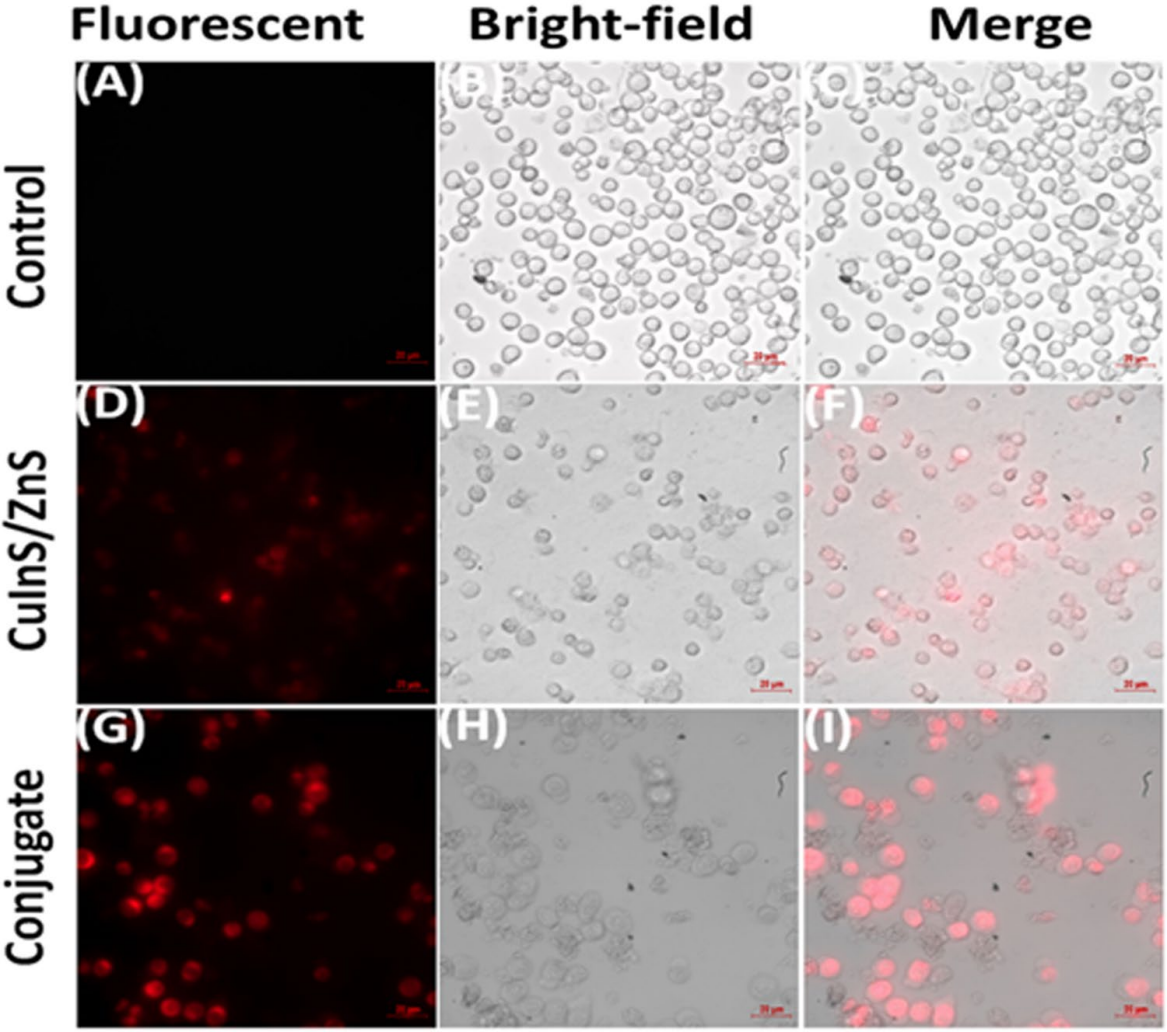
Confocal images of THP-1 cancer cell line under control (**A–C**), CuInS/ZnS QDs (**D–F**) and CuInS/ZnS-mTHPP conjugate (**G–I**) QDs. Gene expressions after exposure of THP-1 human cells to CuInS/ZnS QDs and CuInS/ZnS- mTHPP conjugate.

To demonstrate that the as-synthesized CuInS/ZnS QDs and CuInS/ZnS-mTHPP conjugate could be effective for bio-labelling and can be used to exert drug-efficient photo-biological activity on cells, an uptake of the QDs and conjugate by the treated cells was evaluated. THP-1 cancer cell lines were treated with CuInS/ZnS and CuInS/ZnS-mTHPP for 24 h, and the cell morphology was observed by confocal microscopy. Figure 10 shows a confocal image of THP-1 cells after incubation with CuInS/ZnS QDs and CuInS/ZnS – mTHPP conjugate. The red fluorescence obtained by the QDs and the conjugate can be seen clearly within the cells. This is absent in the control indicating that all the materials are up-taken by the cells. Compared with the cells incubated with CuInS/ZnS QDs only, the cells incubated with the conjugate show better cell internalization. This indicates that the conjugation of the QDs to the free porphyrin enhanced effective intracellular penetration of the conjugate which is important for biological systems thus enabling high activity as seen in Fig. 9b. Similar observation has been reported by Haimov *et al*.^32^, and Zeng *et al*.^33^, for porphyrin conjugated to gold NPs.

### Gene expression study

**Figure 11:**
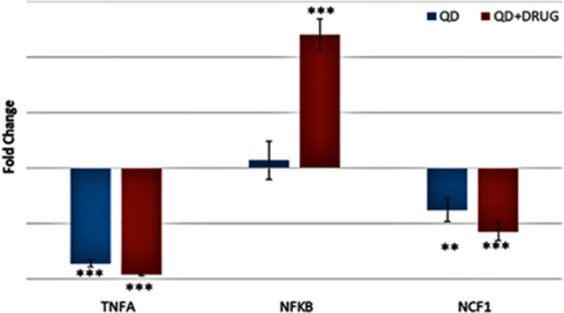
Gene expressions after exposure of THP-1 human cells to CuInS/ZnS QDs and CuInS/ZnS- mTHPP conjugate. Change in the of spectra of (A) DPBF as control, (B) DPBF in the presence of mTHPP and (C) DPBF in the presence of CuInS/ZnS-mTHPP conjugate.

To evaluate variation of gene expression, THP-1 human cancer cells were exposed to CuInS/ZnS QDs and the CuInS/ZnS-mTHPP conjugate for 6 h. Figure 11 presents the gene expression levels of neutrophil cytosolic factor 1 (NCF1), tumour necrosis factor alpha (TNFA), and nuclear factor kappa-light-chain-enhancer of activated B cells (NFKB), which are shown as the fold change relative to the RPL1 ± SE using ANOVA followed by Tukey-Kramer method. A significant increase in NFKB gene expression was observed after exposure to CuInS/ZnS and CuInS/ZnS-mTHPP conjugate. However, the conjugate induced inflammation as NFKB gene was over-expressed upon cell inflammation compare to the QDs. The gene expression study further shows that TNFA and NCF1 were under expressed with slight differences in the CuInS/ZnS and CuInS/ZnS-mTHPP conjugate.

### Singlet oxygen evaluation of CuInS/ZnS, mTHPP and CuInS/ZnS-mTHPP conjugate

**Figure 12:**
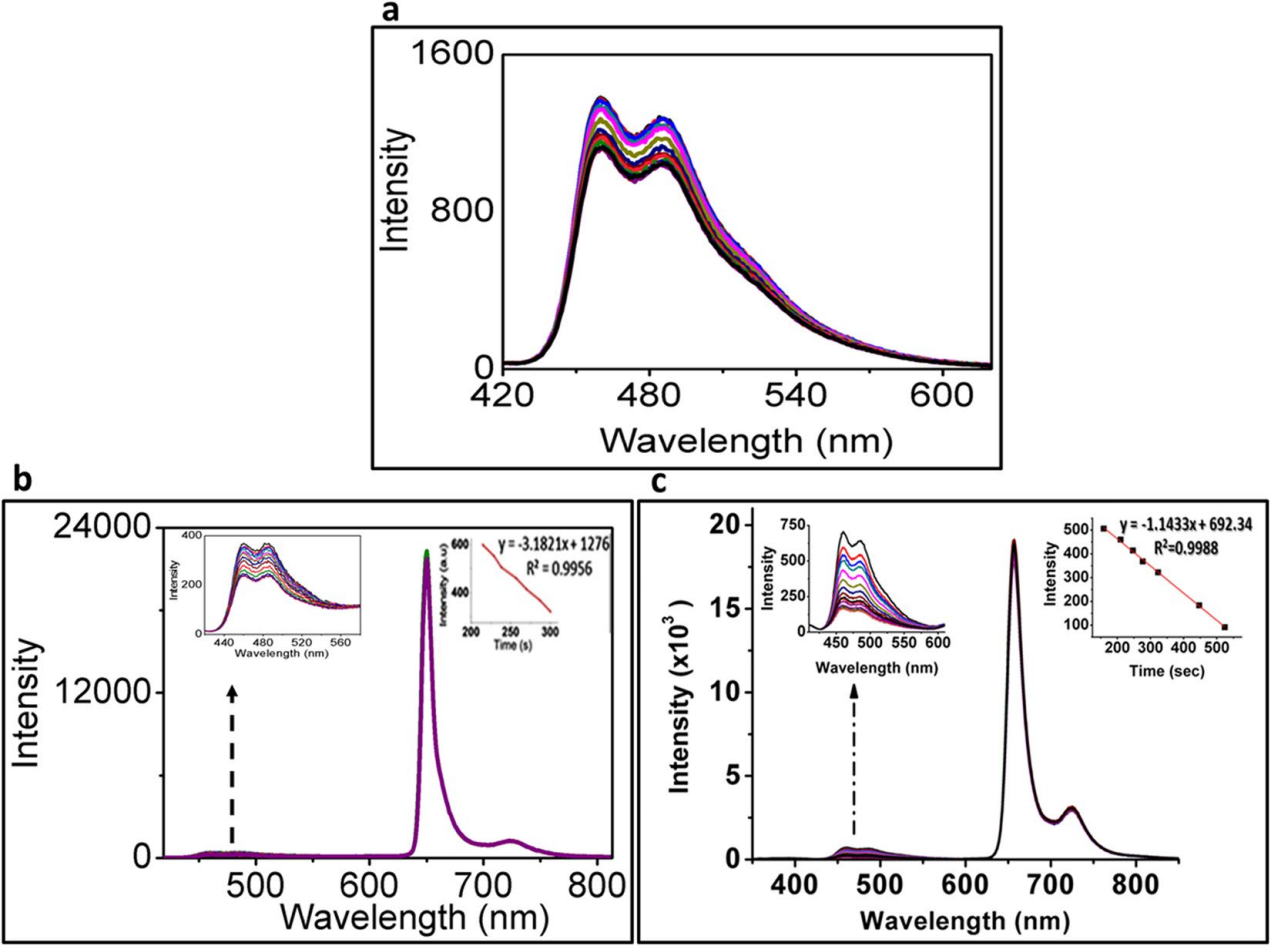
Change in the of spectra of (**a**) DPBF as control, (**b**) DPBF in the presence of mTHPP and (**c**) DPBF in the presence of CuInS/ZnS-mTHPP conjugate.

Excitation of the PS usually leads to the transfer of energy to the surrounding molecular oxygen for the production of singlet oxygen that affects cell viability and cause apoptosis^34^. The singlet oxygen production yield (SOQY) of the mTHPP and CuInS/ZnS-mTHPP conjugate was measured using 1,3-diphenylbenzofuran (DPBP) as oxygen absorber upon irradiation at 657 nm using spectro-fluorophotometry laser light for 365 s. The DPBP traps the produced singlet oxygen and shows a decrease in its fluorescence intensity at 471 nm. Figure 12 shows that the fluorescence intensity of DPBF alone exhibited no appreciable difference at 471 nm under laser irradiation at 657 nm for 365 secs (Fig. 11a), indicating DPBF was stabilized in the mixture solution. On the contrary, a decrease in DPBF intensity (at 471 nm) due to its oxidation as a result of the singlet oxygen generation was observed in the presence of mTHPP (Fig. 11b) and CuInS/ZnS-mTHPP (Fig. 11c). The spectra show high and rapid reduction in the DPBF intensity of the conjugate solution compare to the mTHPP. The SOQY was estimated to be 0.27 and 0.72 for mTHPP and CuInS/ZnS-mTHPP (conjugate) respectively. This indicates that the conjugation of the QDs to the bare porphyrin enhanced its singlet oxygen yield and improve its efficiency by almost three times. This further confirms that Fluorescence Resonance Energy Transfer (FRET) occurred between be the QDs and the porphyrin. The spectral overlap and the close proximity of the mTHPP absorption and CuInS/ZnS fluorescence emission (Supplementary Fig. S3) buttress this. Furthermore, continuous irradiation of mTHPP and the conjugate with light at 657 nm in the absence of DPBF did not exhibit any spectral changes indicating the photo-stability of the as-synthesized materials.

## Conclusion

Glutathione-capped CuInS based QDs were synthesised at large scale using a non-injection hydrothermal method in a household pressure cooker. The QDs were synthesised at different Cu: In ratio’s (1:4 and 1:8) and temperatures (95 °C, 190 °C and 235 °C) with optimised condition at ratio 1.4 and 95 °C. After shell passivation, the CuInS QDs red-shifted from 525 nm to 607 nm and 503 nm to 613 nm at ratio 1:4 and 1:8 respectively. This red shifting after the shell formation reported for the first time for aqueous synthesised ternary CuInS based QDs is attributed to the dominance of quantum confinement effect over cation exchange process. Upon conjugation of the QDs with mTHPP, the conjugate maintained the spectroscopic properties of both the CuInS/ZnS QDs and mTHPP and exhibited good optical properties. Excellent cell viability was achieved by the CuInS/ZnS QDs against normal fibroblast cells indicating its potential use in bio-applications. After conjugation, the cell viability of the conjugate against THP-1 human leaukemia cancer cell line was greatly reduced (‹ 40%) with increased inflammation compared to the QDs alone. In addition, the conjugation of the QDs to the porphyrin improved its cell internalization with improved singlet oxygen yield (three fold) compared to the porphyrin alone. This indicates that the conjugation of the QDs to porphyrin improve its therapeutic effect and it can be used for the development of a better therapeutic agent.

## Materials and Methods

### Chemicals for synthesis

Copper chloride (CuCl_2_), indium chloride (InCl_3_), sodium citrate (Na_3_C_6_H_5_O_7_), L-glutathione (GSH), sodium sulphide (Na_2_S), zinc acetate dihydrate (Zn(O_2_CCH_3_)_2_(H_2_O)_2_), thiourea (CH_4_N_2_S), sodium hydroxide (NaOH), hydrogen chloride (HCl), ethanol (CH_3_CH_2_OH), ethyl acetate (CH_3_COOCH_2_CH_3_), methanol (MeOH) pyrrole, n-hexane, 3-hydroxybenzaldehyde, propionic acid, sodium bicarbonate (NaHCO_3_), methylene blue (MB), 1,3-diphenylbenzofuran (DPBF) and dimethyl sulfoxide (DMSO) were all purchased from Sigma Aldrich, South Africa. The 4-L electric pressure cooker was bought from www.takealot.com. Amphotéricine B-1, DMEM - high glucose, glutamine, SVF, trypsin, Pénicilline streptomycin were purchased from Sigma Life Sciences, France. Isopropanol and chloroform were purchased from Carlo Erba reagents, France. Trizol extract-ALL Rna extraction reagent was purchased from OMEGA bio-tek and WST-1 cell proliferation reagent was purchased from Roche. iScript™ cDNA Synthesis Kit was purchased from Bio-Rad (Marnes-la Coquette, France). All chemicals were used without further purification except for pyrrole which was distilled before it was used.

### Synthesis of CuInS/ZnS QDs

**Figure 13:**
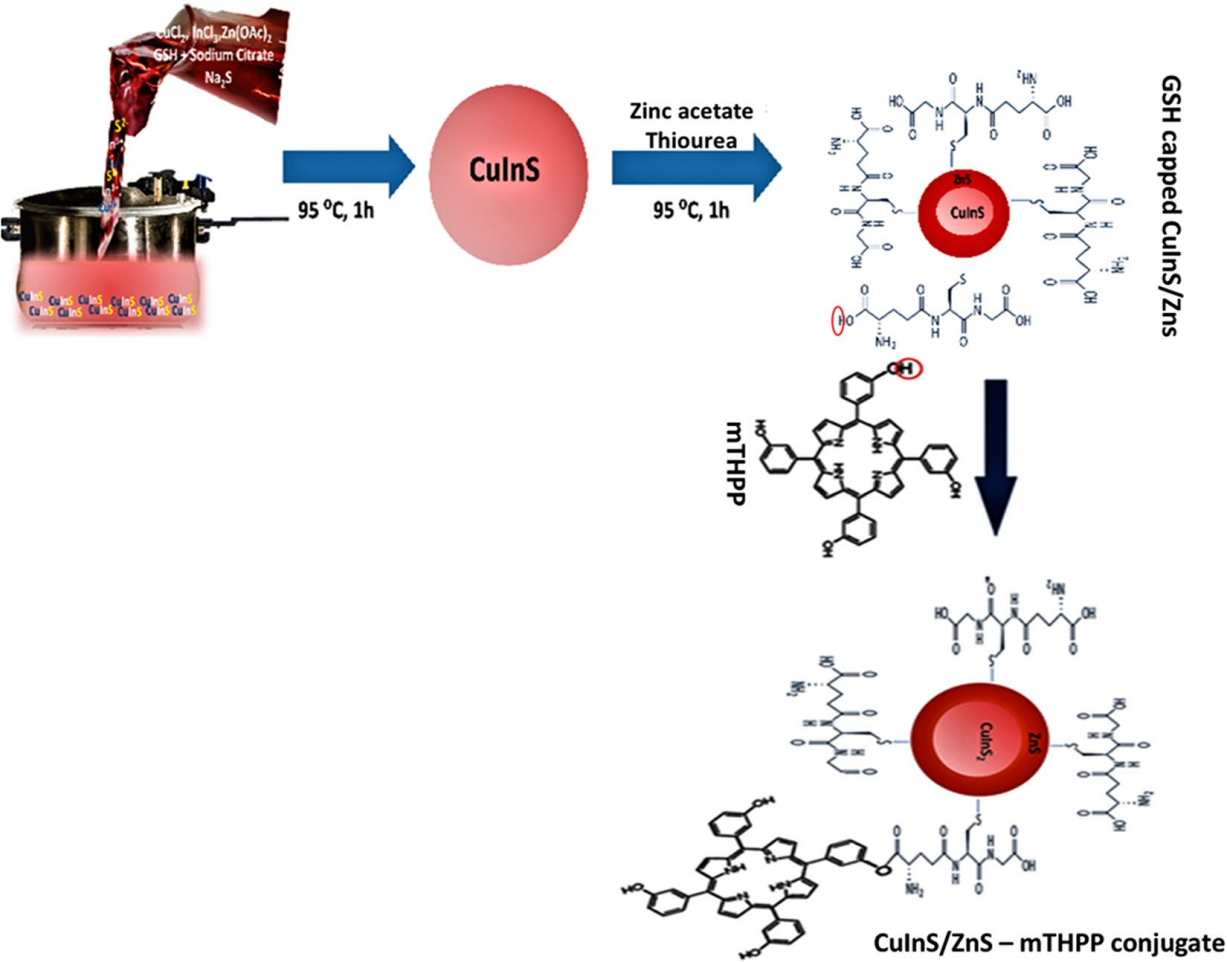
Graphical illustration for the synthesis of CuInS and CuInS/ZnS QDs and the subsequent conjugation of the as-synthesized QDs with mTHPP to form water-soluble CuInS/ZnS QDs – mTHPP conjugate.

CuInS and CuInS/ZnS QDs were synthesized hydrothermally using a commercial kitchen pressure cooker based on our recently reported method with modifications^33^, as shown in Fig. 13. In a typical reaction, CuCl_2_ (0.213 g, 1.26 mmol), InCl_3_ (1.11 g, 5.00 mmol), Na_3_C_6_H_5_O_7_ (5.88 g, 20.14 mmol), GSH (0.276 g, 0.898 mmol) were added to 2.00 L of deionised water under magnetic stirring. This was followed by Na_2_S (1.95 g/50 ml, 25.0 mmol) under magnetic stirring to initiate the reaction. The solution was heated at 95 °C for 1 h to produce CuInS core at Cu: In (1:4) ratio. After 1 h, the pressure cooker was allowed to cool (~86 °C), then, Zn(O_2_CCH_3_)_2_(H_2_O)_2_ (3.52 g/100 ml, 16.0 mmol) and CH_4_N_2_S (1.22 g/100 ml, 16.0 mmol) were added *in-situ* as ZnS precursors. The reaction was further heated for 1 h at 95 °C to form CuInS/ZnS core/shell. The temporal evolution of the optical properties was investigated by following the same procedure for the preparation of the core and core-shell but at 1:8 Cu:In ratio and at different temperatures of 190 °C and 235 °C.

### Synthesis of 5,10,15,20-tetrakis(3-hydroxyphenyl)porphyrin (mTHPP)

The synthesis of mTHPP was carried out according to Rojkiewicz *et al*.^34^ method with modifications. In a typical reaction, 3-hydroxybenzaldehyde (2.50 g, 20.5 mmol) was added to propionic acid (15.0 mL) in a 100 mL round-bottom flask equipped with a magnetic stirring bar and refluxed for 15 minutes at 140 °C. Distilled pyrrole (1.42 mL, 20.5 mmol) was then added quickly to the refluxing mixture and refluxed for an additional 1.5 hours. After the reflux, excess propionic acid was evaporated at 220 °C. The material was cooled to room temperature and neutralized with 225 mL of 5% NaHCO_3_. The precipitated crude porphyrin was washed with chloroform and finally chromatographed on silica gel column with ethyl acetate: n-hexane (2:1, v/v) mixture as an eluent.

### Conjugation of CuInS/ZnS QDs – mTHPP

3.00 mL of CuInS/ZnS (2.50 mg/10.0 ml H_2_0) at Cu: In (1:4) was added to 1.00 mL of mTHPP (0.10 mg/10.0 ml CH_3_OH) followed by mechanical stirring overnight at room temperature. Conjugation of the CuInS/ZnS QDs to the free porphyrin was achieved at mole ratio of 1:3 (CuInS/ZnS (0.0800 mg/ml): mTHPP (0.250 mg/ml)) via an esterification reaction between the hydroxyl groups of the free porphyrin and the hydrogen from the carboxylic group of the glutathione which serves as the capping agent for the QDs as shown in Fig. 13. The conjugate was precipitated out of the solution with ethanol and centrifuged to remove free CuInS/ZnS QDs or mTHPP.

### Characterization

The as- synthesized CuInS/ZnS QDs, mTHPP and conjugate were characterized using; ultraviolet- Visible spectrophotometry (UV–Vis) (Perkin Elmer UV–Vis Lambda 25 spectrometer, (UK); 1 nm slit width), Photoluminescence (PL) (RF-6000, Shimadzu, Japan), Fourier Transform Infrared spectroscopy (FT-IR) (Spectrum two UATR spectrometer, Perkin Elmer, UK), and CFX Connect^TM^ Real-Time system (BIO-RAD Laboratories, Marnes-la-Coquette, France). Transmission electron microscopy (TEM) and Energy Dispersive Spectroscopy (EDS) measurements were carried out using JEOL 2010 operated at 200 kV. X-ray diffraction (XRD) patterns of the as-synthesized QDs were collected by using PANalyticalX’Pert x-ray diffractometer using a monochromatic Cu Kα radiation (λ = 0.15406 nm) at room temperature.

### Cytotoxicity studies of CuInS/ZnS QDs on normal kidney fibroblasts (BHK21)

A (3-(4,5-dimethylthiazol-2-yl)-5-(3-carboxymethoxy-phenyl)-2-(4-sulfophenyl)-2H-tetrazolium) (MTS) was used to determine the *in-vitro* cytotoxicity of the QDs on normal kidney fibroblasts (BHK21) cell line. The cells (1 ×105 cells/ml) were incubated in 96 well plates at 37 °C overnight, with the subsequent addition of the synthesized GSH capped CuInS/ZnS QDs at different concentrations (1.60 μg/ml, 3.10 μg/ml, 6.25 μg/ml, 13.0 μg/ml, 16.0 μg/ml, 31.0 μg/ml, 63.0 μg/ml, 125 μg/ml and 250 μg/ml). The cells were left to incubate for 4 days, whereupon MTS (5.00 μl) was added to the cells. The absorbance values were measured at 490 nm after 1 h, 2 h and 4 h incubation periods, averaged and the viability curves were drawn up.

### Cytotoxicity studies of CuInS/ZnS QDs and CuInS/ZnS-mTHPP conjugate on THP-1 cell line

For the cell viability tests, THP-1 cells (5 × 10 5 cells/well) was seeded into a 96-well plate. After 24 h, the media were replaced with 100 µl of media (DMEM 0% FBS) without phenol red to minimize interactions. The cells were treated with CuInS/ZnS QDs and CuInS/ZnS-mTHPP conjugate at different concentrations (32.0 μg/ml, 63.0 μg/ml, 125 μg/ml and 250 µg/ml) and incubated for 24 h. After treatment and according to manufacturer instructions, 5 µl of WST1 was added and incubated for 2 h at 37 °C. Sample absorbance was measured at 450 nm by using a microplate reader (iMarKTM Microplate Reader, (BIO-RAD Laboratories, Marnes- la-Coquette, France). The mean absorbance of non-exposed cells served as the reference value for calculating 100% cellular viability.

### Gene expression study of CuInS/ZnS QDs and CuInS/ZnS-mTHPP conjugate on THP-1 cell line

The THP-1 cells were treated with 63 µg/ml of CuInS/ZnS QDs and CuInS/ZnS-mTHPP conjugate for 6 h after-which the cells were lysed by adding 1.0 ml of Trisol, followed by the addition of 200 µl of chloroform. The samples were then centrifuged for 15 min and 500 µl of isopropanol was added to the supernatant and then washed with ethanol at 80 °C. The samples were incubated for 10 min at 60 °C to eliminate ethanol, followed by dilution with RNase-free water. RNA purity was assessed using the BioSpec-nano Spectrophotometer (SHIMADZU, Japan). RNA degradation of the material was analyzed using RNA 6000 Nano Reagents Kit on Bioanalyzer™ 2100 (Agilent Technologies, Germany). For cDNA conversion, the iScript™ cDNA Synthesis Kit was used according to manufacturer’s instructions. A mixture consisting of diluted cDNA, iQ™ SYBR Green® Supermix and primer (Table 1) for each gene was analyzed by a CFX ConnectTM Real-Time system followed by the thermal profile settings: 95 °C, 5 min; 95 °C, 1 s followed by 60 °C, 1 min for 40 cycles. Gene expression levels were normalized by comparison to ribosomal protein L13 (RPL13) used as a reference gene for THP-1. Fold changes (FC) of gene expression were calculated by the 2 − ΔΔCt method (Peirson *et al*. 2003).

**Table 1:** Primers used for gene expression analysis in NR 8383 rat cells.

Functional class	Gene	Sequence
	RPL13 (internal control)	F: 5′CCCTCCACCCTATGACAAGA-3′ R:5′GGTACTTCCACCCGACCTC-3′
Oxidative stress	NCF1	F:5′CTTGTAATTCCCGCATTGCT-3′ R:5′-GCCTCGTATGTCTTGATGC-3′
Inflammation	TNFA	F:5′-TAGCCCATGTTGTAGCAAACC-3′ R:5′GATCGGCAGAGGAGGTTGA-3′
Inflammation	NFKB	F:5′TTCGGAACTGGGCAAATGTT-3′ R:5′-ACACGTAGCGGAATCGAAAT-3′

### Singlet oxygen quantum yield of mTHPP and CuInS/ZnS-mTHPP conjugate

Singlet oxygen generation was determined by Adarsh *et al*.^35–39^, method. Briefly, 0.10 mL DPBF solution (prepared by dissolving 0.200 mg DPBF in 10.0 mL DMSO) was diluted to 5.10 mL using DMSO. Then, 1:1 mixture of DPBF and mTHPP and 1:1 mixture of DPBF and CuInS/ZnS – mTHPP conjugate were irradiated at 657 nm using spectro-fluorophotometry laser light for 365 s. The decrease in the intensity of the DPBF was monitored at 471 nm. The singlet oxygen quantum yield (SOQY) was calculated against methylene blue as a standard (SOQY = 0.52) using the following formula:

1 $$SOQY=\left(\frac{{m}_{c}}{{m}_{ref}}\right)\times \left(\frac{{A}_{ref}}{{A}_{c}}\right)\times SOQ{Y}_{ref}$$

where SOQY and SOQYref are the singlet oxygen quantum yield of the sample (mTHPP or Conjugate) and the reference (methylene blue, 0.520), mc and mref are the slope of the sample and reference, Aref and Ac are absorbance’s of the reference and the sample at irradiation wavelength.

## Supplementary information

Supplimentary dataset.

## Data Availability

All data is available upon request.
