# Supplementary material for: Cytotoxicity, fluorescence tagging and gene-expression study of CuInS/ZnS QDS - meso (hydroxyphenyl) porphyrin conjugate against human monocytic leukemia cells
Source: Sci Rep. 2020 Mar 18;10:4936. doi: 10.1038/s41598-020-61881-8 (PMC7080734; doi:10.1038/s41598-020-61881-8)
Supplement: Supplementary file 1 — Supplimentary dataset. [file 41598_2020_61881_MOESM1_ESM.docx]

**Cytotoxicity, fluorescence tagging and gene-expression study of CuInS/ZnS QDS - meso (hydroxyphenyl) porphyrin conjugate against human monocytic leukemia cells**

Ncediwe Tsolekile ^1,2,3^, Sara Nahle ^4^, Nkosingiphile Zikalala ^1,2^, Sundararajan Parani ^1,2^, El Hadji Mamour Sakho ^1,2^, Olivier Joubert ^4^, Mangaka C Matoetoe ^3^, Sandile P Songca ^5^ and Oluwatobi S Oluwafemi *^1,2^

^
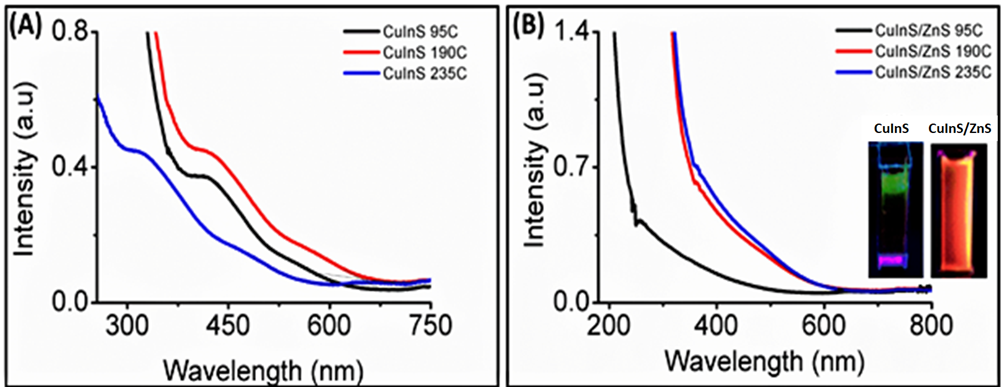
^

**S1:** UV of (A) CuInS QDs core and (B) CuInS/ZnS QDs core/shell synthesized at Cu:In (1:4) at different reaction temperatures (insert; CuInS and CuInS/ZnS under UV lamp at 325 nm)


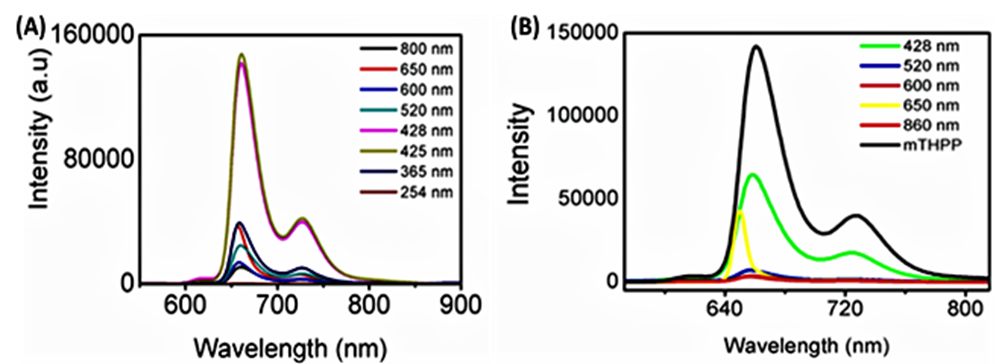


**S2:** Fluorescence spectra of (A) mTHPP and (B) CuInS/ZnS-mTHPP conjugate at different excitation wavelengths.


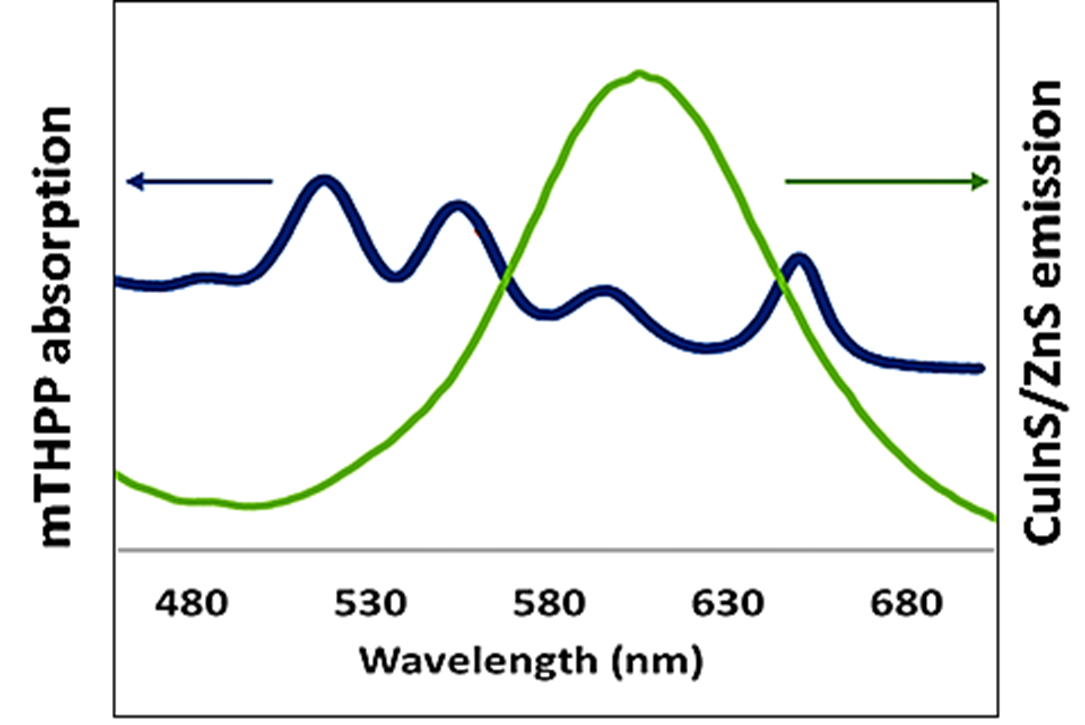


**S3:** Spectral overlap of mTHPP absorption and CuInS/ZnS fluorescence emission
